# IL-4/IL-13-Dependent and Independent Expression of miR-124 and Its Contribution to M2 Phenotype of Monocytic Cells in Normal Conditions and during Allergic Inflammation

**DOI:** 10.1371/journal.pone.0081774

**Published:** 2013-12-16

**Authors:** Tatyana Veremeyko, Shafiuddin Siddiqui, Ilya Sotnikov, Amanda Yung, Eugene D. Ponomarev

**Affiliations:** 1 School of Biomedical Sciences, The Chinese University of Hong Kong, Shatin, New Territories, Hong Kong; 2 Center for Neurologic Diseases, Brigham and Women’s Hospital, Harvard Medical School, Boston, Massachusetts, United States of America; Virginia Commonwealth University, United States of America

## Abstract

Monocytic cells exhibit a high level of heterogeneity and have two distinct modes of their activation: 1) classical M1 path associated with inflammation and tissue damage, and 2) alternative M2 path. Although it has been demonstrated that M2 macrophages play an important role in the regulation of the allergic immune responses, tissue maintenance and repair, little is known about the mechanisms that determine the M2 phenotype. We have previously shown that miR-124 is expressed in microglia that exhibit the M2 phenotype and overexpression of miR-124 in macrophages resulted in downregulation of a number of M1 markers (MHC class II, CD86) and up-regulation of several M2 markers (Fizz1, Arg1). We further investigated whether the polarization of macrophages towards the M2 phenotype induced miR-124 expression. We found that exposure of cells to IL-4 and IL-13 resulted in the upregulation of miR-124 in macrophages. We also demonstrated that IL-4 induced expression of three miR-124 precursor transcripts with predominant expression of pri-miR-124.3, suggesting regulation of miR-124 expression by IL-4 on a transcriptional level. Expression of miR-124 in microglia did not depend on IL-4 and/or IL-13, whereas expression of miR-124 in lung resident macrophages was IL-4 and IL-13-dependent and was upregulated by systemic administration of IL-4 or during allergic inflammation. Upregulation of several M2 markers (CD206, Ym1) and downregulation of the M1 markers (CD86, iNOS, TNF) in M2-polarized macrophages was abrogated by a miR-124 inhibitor, suggesting that this microRNA contributed to the M2 phenotype development and maintenance. Finally we showed that human CD14^+^CD16^+^ intermediate monocytes, which are found in increased numbers in patients with allergies and bronchial asthma, expressed high levels of miR-124 and exhibited other properties of M2-like cells. Thus, our study suggests that miR-124 serves as a regulator of the M2 polarization in various subsets of monocytic cells both *in vitro* and *in vivo*.

## Introduction

Monocytic cells represent a complex and heterogeneous population of cells that include tissue resident macrophages nested in various tissues including the central nervous system (CNS), peritoneum, lungs, liver, and adipose tissue [1]. In addition to the tissue resident macrophages, there is a mobile population of monocytes circulating in the peripheral blood that have an ability to transmigrate into tissues during inflammation and differentiate to the inflammatory macrophages [2]. It was shown that under homeostatic conditions circulating monocytes could also transmigrate into tissues to replenish the pool of the tissue resident macrophages [2]; however it was recently suggested that the reservoir of the tissue macrophages is supplied by the local proliferation of the resident cells rather than the egress of monocytic cells from the blood [3]. The circulating monocytes appeared to be also a heterogeneous population of cells, especially in humans, where three distinct subsets were identified: the classical CD14^++^CD16^−^, the intermediate CD14^+^CD16^+^, and the non-classical CD14^low^CD16^+^ monocytes [4], [5].

There are two major pathways of the macrophage activation required for their main functional activities: the classical M1 pathway and the alternative M2 pathway [1], [6]. The classical pathway gives rise to the M1 macrophages during an immune response to infection and is mediated by the Th1 cytokine IFN-γ and the toll-like receptor agonists, such as LPS. The classically activated M1 macrophages express and secrete large amounts of potent pro-inflammatory mediators such as TNF and reactive oxygen species (such as NO) that are associated with substantial tissue damage. Moreover, the M1 macrophages express high levels of MHC class II and, co-stimulatory molecules, such as CD86, which are important for the activation and stimulation of CD4 T cells. The alternatively activated M2 macrophages are induced by the Th2-derived cytokines IL-4 or IL-13 (IL-4/IL-13-induced phenotype is also called the “M2a” subset of M2 population) and to some extent by the cytokines produced by Tregs, such as IL-10 or TGF-β (IL-10/TGF-β-induced phenotype is also called “M2b” subset of M2 population). The M2 macrophages produce a number of factors that are responsible for the anti-parasitic response and promote tissue repair such as Fizz1, Ym1, Arg1, Mannose Receptor (MR or CD206) as well as anti-inflammatory, regulatory, and B cell-stimulating factors such as TGF-β1, IL-4, and IL-10 [6], [7].

In our previous studies we investigated the phenotype and function of the CNS-resident macrophages, which are also referred to as microglia. We found that microglia in the normal CNS expressed a number of the M2 markers, including Fizz1, Ym1, IL-10 and IL-4 [8]. It was recently demonstrated that peritoneal, pleural, splenic, lung, liver and adipose tissue resident macrophages also exhibit properties of the M2-like cells by expressing several M2 markers including CD206, Fizz1 and Arg1 [6], [9]. This indicates that tissue resident macrophages often exhibit properties of M2 cells under normal physiological conditions; however it is unclear how this phenotype is induced and maintained.

Allergic airway and lung inflammation, a hallmark of bronchial asthma, represents a complex pathological condition that is characterized by bronchial hyper-responsiveness, airway eosinophilia, and macrophage activation. It is known that IgE antibodies recognizing a particular allergen contribute to the tissue inflammation by triggering the release of the pro-inflammatory substances from mast cells (e.g. histamine, prostaglandins) culminating in the appearance and accumulation of pro-inflammatory, as well as Th2 cytokines IL-4 and IL-13 [10]. The ovalbumin (OVA)-induced allergic lung inflammation is a widely used mouse model of asthma that recapitulates the airway eosinophilia, pulmonary inflammation and elevated levels of IgE antibodies frequently found in patients with allergic bronchial asthma [11].

MicroRNAs are a family of short (22–23 nucleotides) non-coding RNAs that are involved in the epigenetic regulation of gene expression by controlling the mRNA translation and/or turnover [12]. MicroRNA function is crucial for many physiological processes including cell proliferation, apoptosis and differentiation [13]. It was recently demonstrated that miRNAs are also operative in macrophage polarization [7], [12], [13]. For example, miR-155 and miR-146a are associated with M1 phenotype and are up-regulated by the M1-inducing stimuli such as IFN-γ and LPS [7], [12], [13]. Recent study suggests that miR-223 contributes to the development of the M2 phenotype in macrophages residing in the adipose tissue [7], [14]. We have previously demonstrated that in contrast to the mouse macrophages in the periphery, microglia expressed high levels of miR-124, but not miR-223 [15], and this microRNA contributed to the M2 phenotype in these cells, suggesting the role of miR-124 in polarization of CNS-resident macrophages [13], [15]. However, it was not clear from our previous studies whether miR-124 serves as a universal regulator of M2 macrophage polarization or its function was restricted to the microglial cells in the CNS only.

In this study we investigated how miR-124 contributes to the phenotype of various tissue subsets of the M2 macrophages *in vitro* and *in vivo*. We found that exposure to the Th2 cytokines IL-4 and IL-13 but not the anti-inflammatory cytokines IL-10, TGF-β or the M1-inducing stimuli, such as IFN-γ/LPS, resulted in miR-124 expression in the bone marrow (BM) derived and peritoneal macrophages, as well as in a macrophage cell line RAW 264.7. The expression of miR-124 in the microglia did not require the IL-4/IL-13 receptors and STAT6 signaling pathways, whereas in the lung macrophages, miR-124 was expressed at a low level in an IL-4/IL-13 dependent fashion and was substantially up-regulated during the OVA-induced allergic lung inflammation. We also found that miR-124 was essential for the up-regulation of CD206 and Ym1, and downregulation of CD86, iNOS (NO producing enzyme) and TNF in the M2-polarized macrophages. Finally, we demonstrated that the subset of human intermediate CD14^+^CD16^+^ monocytes, which is associated with asthma progression and allergic inflammation [16], [17], expressed high levels of miR-124. Thus, our study demonstrates that miR-124 contributes to the development of the M2 phenotype in various types of monocytic cells of different origins and high level of expression of this microRNA in lung macrophages is associated with allergic lung inflammation.

## Results

### IL-4 and IL-13 Upregulate miR-124 in Macrophages on a Transcriptional Level

**Figure 1:**
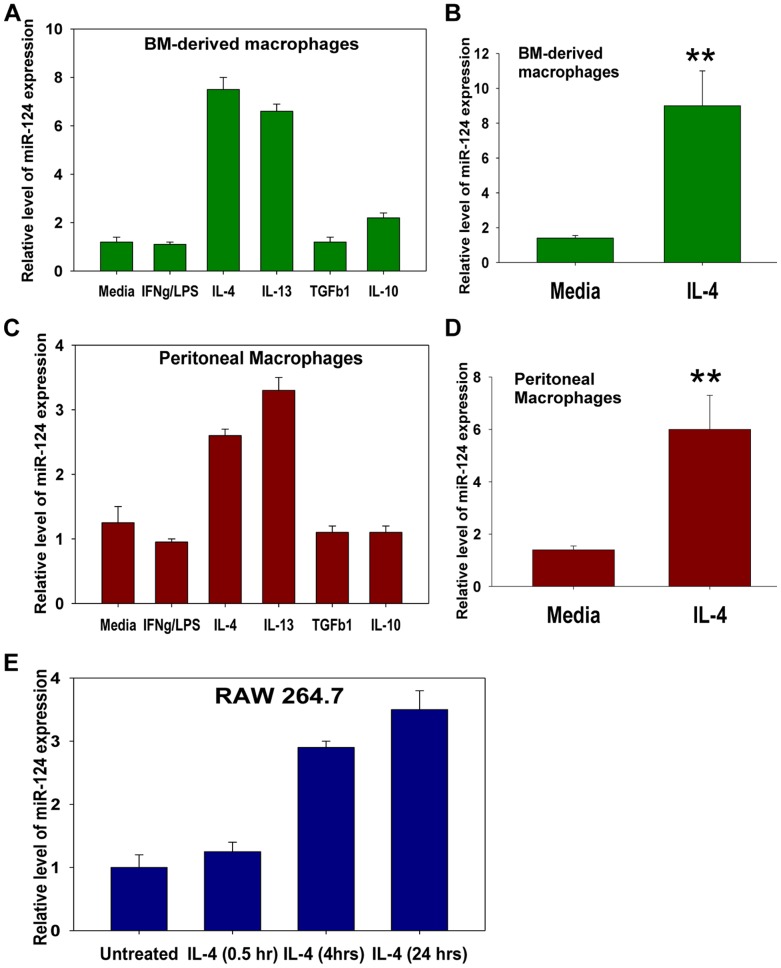
Analysis of miR-124 expression in macrophages polarized *in vitro* towards M1 (IFN-γ/LPS), M2a (IL-4, or IL-13), or M2b (TGF-β1, or IL-10). Bone marrow (BM) derived cultured macrophages (**A, B**), *ex-vivo* isolated CD11b^+^F4/80^+^ peritoneal macrophages (**C, D**), or macrophage cell line RAW 264.7 (**E**) were incubated with media alone (Media), or IFN-γ and LPS (IFN-γ/LPS), or IL-4, or IL-13, or TGF-β1, or IL-10 for 24 hours, after which the expression of miR-124 was measured by the real-time RT-PCR as described in *Materials and Methods*. The data is representative of eight (**A, B**), six (**C, D**) or five (**E**) separate experiments; mean ± S.E. of triplicate is shown in (**A**, **C**, **E**). Mean ± S.E. of eight separate experiments is shown in (**B**) and mean ± S.E. of six separate experiments is shown in (**D**) (**p<0.01).

We have previously found that transfection of macrophages with miR-124 resulted in downregulation of the M1-associated genes, such as TNF, MHC class II, and CD86, while at the same time upegulating the M2-associated genes Fizz1, Arg1, and TGF-β1 [15]. Thus, we hypothesized that miR-124 promotes the M2 polarization. To test our hypothesis we investigated factors that upregulate miR-124 in macrophages and found that exposure to IL-4 and IL-13 (M2a inducers) but not M1-inducing IFN-γ and LPS, or anti-inflammatory cytokines IL-10 or TGF-β1 (M2b inducers) resulted in upregulation of miR-124 in the cultured BM-derived and *ex-vivo* isolated peritoneal macrophages. Thus miR-124 appears to be a marker of the M2a cells (**Fig. 1A**). The observed effect of treatment with IL-4 or IL-13 was detected at a wide range of IL-4 or IL-13 concentrations (10–200 ng/ml; not shown) and was stronger for the BM-derived macrophages resulting in a 6–12-fold upregulation of miR-124. However, there were interexperimental variations, depending on the initial assay conditions, in BM-derived macrophages cultured in the presence of M-CSF. Statistical analysis of eight separate experiments for IL-4 treated vs. untreated BM-derived macrophages is shown in **Fig. 1B** **.** The effect of IL-4 or IL-13 on the *ex-vivo* isolated peritoneal macrophages or the macrophage line RAW 264.7 was less prominent, resulting in a 2–8-fold upregulation of miR-124, but these results were more consistent in the experimental repeats (**Fig. 1C, E**). Statistical analysis of six separate experiments for IL-4 treated vs. untreated peritoneal macrophages is shown in **Fig. 1D** **.** We explained the observed differences by the various baseline “M0 states” of activation of BM-derived and peritoneal macrophages. For example, the peritoneal macrophages from B6 mice at their M0 state expressed high levels of IL-4 when compared to the BM-derived macrophages of the same mouse strain or the *ex-vivo* isolated adult microglia, which was shown to be of the M2 phenotype [8] (**Fig. S1A**). Thus, peritoneal macrophages already possess an M2-like phenotype and produce endogenous IL-4 that may act as an autocrine factor; therefore these cells may be less responsive to the exogenous IL-4 exposure. In contrast to the *ex-vivo* isolated peritoneal macrophages, cultured BM-derived macrophages from B6 mice expressed TNF at their M0 state (**Fig. S1B**), demonstrating M1-like properties. The spontaneous expression of TNF in the BM-derived macrophages varied from one experiment to another influencing their responsiveness to IL-4 and the extent of the IL-4-induced M2 phenotypical markers (e.g. Fizz1, Arg1) and miR-124 expression (not shown).

Since the induction of miR-124 in macrophages by IL-4 was highly consistent in the peritoneal macrophages as well as the macrophage cell line RAW 264.7, we further investigated the kinetics and molecular mechanisms of miR-124 up-regulation by IL-4 in the RAW 264.7 model system. This macrophage cell line provided us with the desired level of reproducibility and sufficient cell numbers needed for RNA isolation and analysis. We found that miR-124 was upregulated as early as at 4 hours of RAW 264.7 incubation with IL-4 in cell culture (**Fig. 1C**). We further investigated particular molecular mechanisms of how miR-124 was upregulated in the macrophages treated with IL-4. In theory IL-4 could induce miR-124 on a transcriptional level or by enhancing the processing of this miRNA as was shown previously for other cell types [18], [19]. If IL-4 induces miR-124 on the transcriptional level, then longer precursor transcripts (pri-miRNA) should be detected in the IL-4 treated macrophages. Indeed, we found that IL-4 induced the expression of all three known precursors for miR-124: pri-miR-124.1, pri-miR-124.2 and pri-miR-124.3. The expression of pri-miR-124.3 was detected as early as at 30 minutes (0.5 hour) after exposure of the macrophages to IL-4 and was still observed 24 hours later. The expression of two other transcripts, pri-miR-124.1 and pri-miR-124.2, was transient and was detected less frequently between experiments (**Table 1**). To conclude, we found that IL-4 is capable of inducing the miR-124 expression in macrophages *in vitro* on a transcription level.

**Table 1:** Analysis of kinetics of expression of the miRNA-124 precursor transcripts pri-miRNA-124.1, pri-miRNA-124.2 and pri-miRNA-124.3 in RAW264.7 cells treated with IL-4^1^.

			Treated with IL-4		
Experiment #	pri-miRNA	Untreated	0.5 h	4 h	24 h
**1**	Pri-miR-124-1	**−** 2	**−**	**+** 3	**−**
	Pri-miR-124-2	**−**	**−**	**+**	**−**
	Pri-miR-124-3	**−**	**+**	**+**	**−**
**2**	Pri-miR-124-1	**−**	**−**	**−**	**−**
	Pri-miR-124-2	**−**	**−**	**−**	**−**
	Pri-miR-124-3	**−**	**+**	**+**	**+**
**3**	Pri-miR-124-1	**−**	**+**	**+**	**−**
	Pri-miR-124-2	**−**	**−**	**−**	**−**
	Pri-miR-124-3	**−**	**+**	**+**	**+**
**4**	Pri-miR-124-1	**−**	**−**	**+**	**−**
	Pri-miR-124-2	**−**	**−**	**+**	**−**
	Pri-miR-124-3	**−**	**−**	**+**	**−**

*in Materials and Methods*. Data of four experiments performed in triplicates for each precursor is shown.^1^ The cells were treated with 50 ng/ml of IL-4 and harvested at indicated time points after adding of IL-4. RNA was isolated and expression of pri-miR-124.1, pri-miR-124.2 or pri-miR-124.3 was analyzed by real-time RT PCR as described

“−”) if no product was detected after more than 40 cycles of amplification.^2^ Expression was considered negative (

“+”) for C_T_<35 cycles.^3^ Expression was considered positive (

### Expression of miR-124 in Microglia is IL-4/IL-13-independent

In the following experiments we investigated whether IL-4 or IL-13 was required to induce the expression of miR-124 in macrophages *in vivo*. We have previously shown that microglia in the normal CNS exhibited properties of the M2-type macrophages [8] and expressed high levels of miR-124 but not the M1 phenotype associated miR-155 or another reported M2-associated miR-223 [15]. During the course of the Th1-mediated autoimmune inflammation (i.e., experimental autoimmune encephalitis) the expression of miR-124 declined [15], while the CNS-derived IL-4 was shown to be important for the induction of several M2 markers in the microglia [8]. Thus the CNS-derived IL-4 and IL-13 could potentially induce miR-124 expression in the microglia of the normal CNS. To investigate whether IL-4 and IL-13 induce the expression of miR-124 in the CNS, we compared the levels of miR-124 expression in the microglia isolated from the wild type (WT), STAT6 (Signal transducer and activator of transcription 6 that is downstream of IL-4 and IL-13 receptors)-, or IL-4-, and IL-4/IL-13 receptor common α-chain (IL-4/13R or IL-4Rα)-deficient mice. We found that in the CNS of healthy STAT6-, IL-4- and IL-4/13R-deficient mice microglia had typical non-activated CD45^low^MHC class II negative phenotype similar to that of WT controls (not shown) and did not have decreased levels of miR-124 when compared to the WT controls of the same strains (**Fig. S2**). We have concluded that IL-4 and IL-13 did not contribute to the expression of miR-124 in the microglia of the CNS *in vivo*. In our previous experiments we demonstrated that co-culture of macrophages with a neuronal line resulted in the up-regulation of miR-124 [15]. Thus we proposed that certain neuronal factors exist besides IL-4 or IL-13 that have an ability to upregulate the miR-124 expression in the microglia of the CNS [13]. Thus, miR-124 expression in the CNS-resident microglia is IL-4/IL-13 independent.

### Expression of miR-124 in Normal Lung Resident Macrophages is IL-4/IL-13-dependent

**Figure 2:**
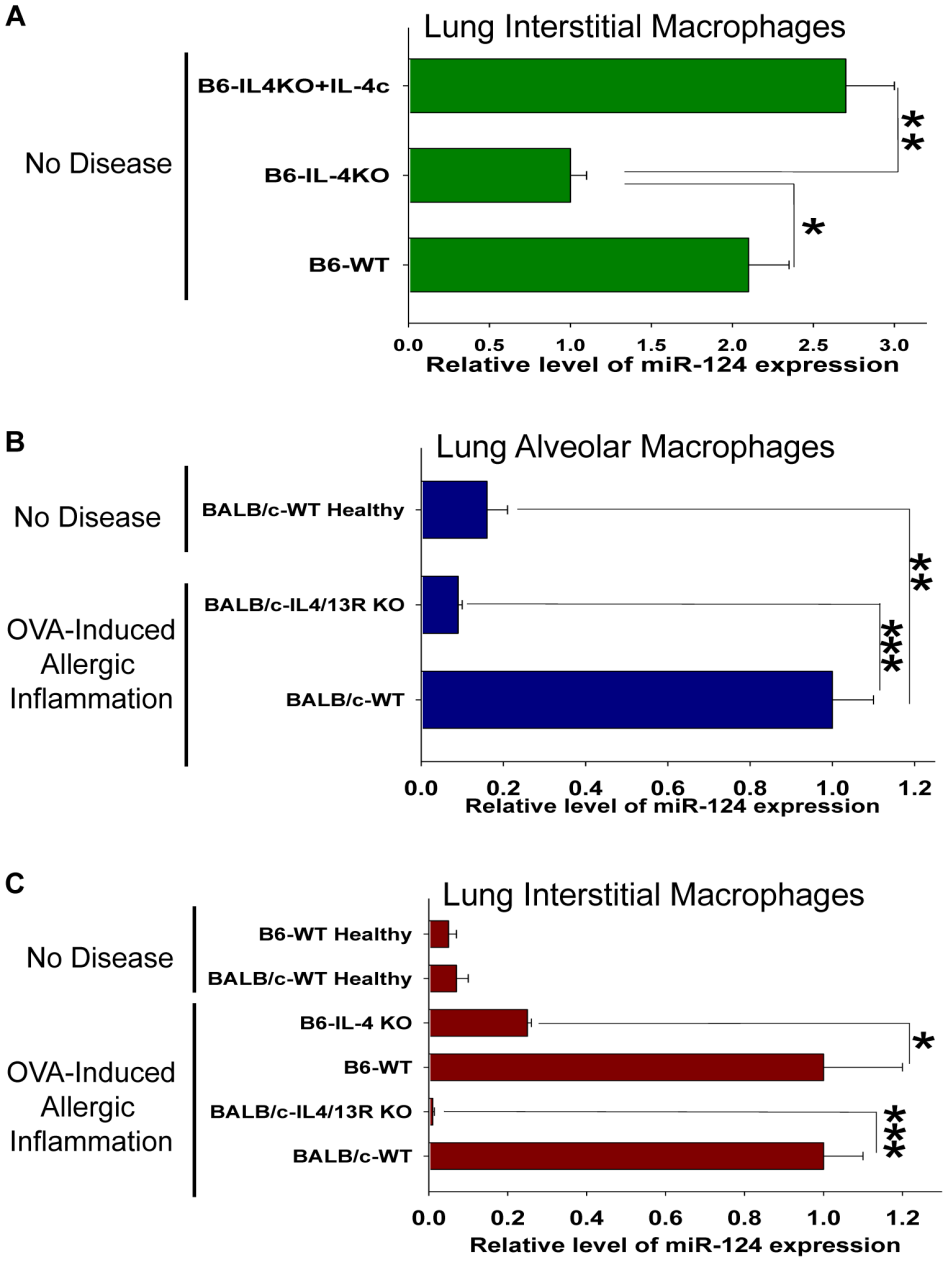
Analysis of the miR-124 expression in lung macrophages in the baseline conditions or during the OVA-induced allergic lung inflammation. (**A**) Lung interstitial macrophages were isolated from the unmanipulated B6-WT mice, B6-IL-4 deficient mice, or B6-IL-4 deficient mice in which IL-4c was systemically administered as described in *Materials and Methods* and the expression of miR-124 was measured as in Fig. 1. (**B**) Lung alveolar macrophages were isolated from the unmanipulated BALB/c-WT healthy mice, or BALB/c-WT and BALB/c-IL-4/13R deficient mice with OVA-induced allergic lung inflammation and the expression of miR-124 was analyzed. (**C**) Lung interstitial macrophages were isolated from healthy B6-WT and healthy BALB/c-WT vs. B6-WT and B6-IL-4 deficient mice, or BALB/c-WT and BALB/c-IL-4/13R deficient mice with an OVA-induced allergic lung inflammation and expression of miR-124 was analyzed. In (**A–C**), Mean ± S.E. of 4–6 individual animals is shown (*, p<0.05; **, p<0.01; ***, p<0.005).

Since we found that IL-4 or IL-13 did not induce miR-124 in the microglia of the CNS (**Fig. S2**), we investigated the expression of miR-124 under the influence of IL-4 or IL-13 in the resident macrophages of other tissues such as lungs. We selected lung macrophages since they exhibit an M2 phenotype similarly to testicular macrophages [20], and the lung is the organ harboring the allergic inflammation in human pathologies such as asthma, mediated by IL-4 and IL-13 [11], [17]. Although the expression of miR-124 in the lung resident (interstitial) macrophages was lower than in the microglia, it was still detected in WT B6 mice. Expression of miR-124 in IL-4 deficient (or in IL-4/13R deficient mice; not shown) was 2–4 fold lower compared to WT mice (**Fig. 2A**). We hypothesized that endogenous IL-4 contributed to the miR-124 expression in the lung resident macrophages under baseline conditions. To confirm this, we injected intravenously IL-4 into IL-4 deficient mice that lack endogenous IL-4, and registered a 2.7-fold upregulation of miR-124 in the lung tissue macrophages which reached the level comparable to WT mice with endogenous IL-4 (**Fig. 2A**). This finding suggests that endogenous IL-4 mediates miR-124 expression in the lung macrophages *in vivo*.

### Expression of miR-124 is Substantially Upregulated in the Lung Macrophages during an Allergic Inflammation

To investigate the miR-124 dynamics during inflammation, we used the mouse asthma model, also known as “OVA-induced allergic lung inflammation”. It was demonstrated that similar to human asthma, this experimental disease is mediated by IL-4 and IL-13 cytokines [11]. We compared the level of the miR-124 expression in the alveolar macrophages sorted by FACS from the BAL cells of WT vs. IL-4/13R deficient mice with the OVA-induced allergic lung inflammation. As shown in **Fig. 2B**, the expression of miR-124 in alveolar macrophages was upregulated in WT but not in IL-4/13R deficient mice during the course of the allergic inflammation. It was previously demonstrated that the IL-4/13R deficient mice are resistant to the OVA-induced lung inflammation [21], consistent with the fact that in our experiments the level of the miR-124 expression in the macrophages of IL-4/13R deficient mice challenged with OVA was even lower than in healthy mice (**Fig. 2B**; Healthy WT mice). Finally we found that miR-124 was expressed at a high level during the allergic inflammation in the lung interstitial macrophages when compared to healthy mice of B6 and BALB/c strains (**Fig. 2C**). The presence of miR-124 was barely detectable in the lung resident (interstitial) macrophages from the IL-4/13R deficient mice during the allergic inflammation and was reduced 5-fold in IL-4 deficient mice when compared to WT controls of the same strains (**Fig. 2C**). Thus the expression of miR-124 is highly upregulated in the IL-4/IL-13 dependent manner in the lung macrophages during the allergic inflammation.

### M1 Macrophages are Resistant to Induction of M2 Phenotype and Expression of miR-124

**Figure 3:**
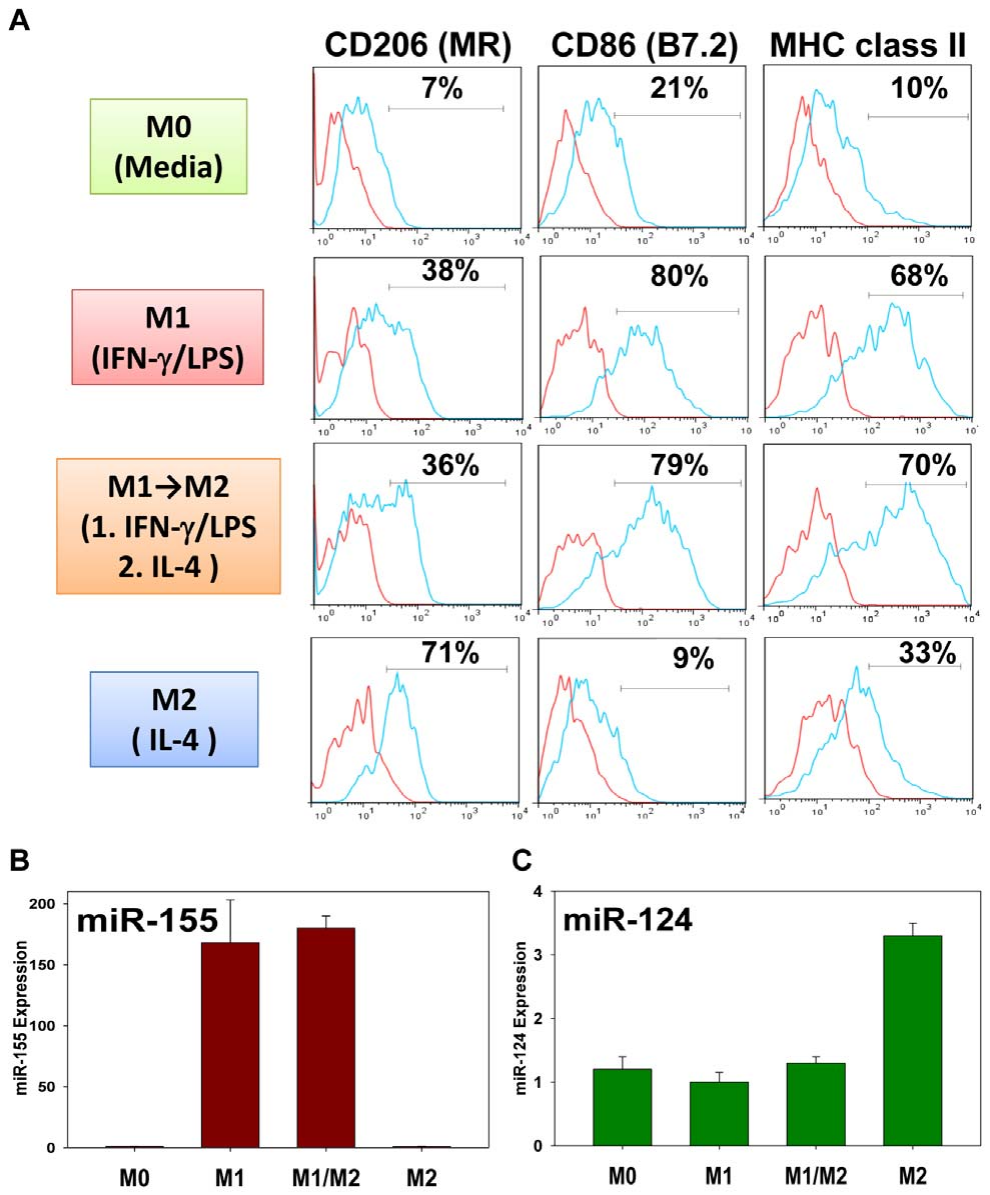
Treatment of M1 macrophages with IL-4 did not result in upregulation of miR-124 and conversion of M1 cells into M2. Peritoneal CD11b^+^F4/80^+^ macrophages were isolated and left unpolarized (*M0*) or polarized towards *M1*, or *M2*, or first polarized towards M1, and after 24 hours, washed and polarized towards M2 (*M1→M2* or *M/M2*) as described in *Materials and Methods*. After which, the cells were stained for CD206, CD86 and MHC class II and analyzed by FACS (**A**), or used for RNA isolation and analysis of expression of miR-155 (**B**) or miR-124 (**C**) by real-time RT-PCR. One representative experiment of three is shown in (**A–C**); mean ± S.E. of triplicate is shown in (**B**, **C**).

We next investigated whether miR-124 could be induced by IL-4 in M1 macrophages and whether M1 macrophages could be converted into M2 in miR-124 dependent manner. To test this we polarized macrophages towards M1 for 24 hours, washed them by changing media several times, and then incubated cells with IL-4 for next 24 hours. Unpolarized (M0), M1, and M2 polarized macrophages were used as a proper reference controls. We found that M1 macrophages treated with IL-4 neither downregulate M1 markers nor upregulate M2 markers and exhibited CD206^low^MHC class II^hi^CD86^hi^ phenotype (**Fig. 3A, ***M* *1→M2*) that resembled M1 (**Fig. 3A, ***M* *1*). On the other hand M2 macrophages upregulated CD206 and had low level of CD86 when compared to M0 (**Fig. 3A, ***M* *0*). M2 macrophages exhibited typical CD206^hi^MHC class II^int^CD86^lo^ phenotype (**Fig. 3A, ***M* *2*). In addition, M1-specific miR-155 was highly upregulated in M1 macrophages (**Fig. 3B, ***M* *1*) and stayed at a very high level in M1 macrophages treated with IL-4 (**Fig. 3B, ***M* *1/M2*) when compared to M0 and M2 macrophages that expressed very low levels of miR-155 (**Fig. 3B, ***M* *0* and *M2*). We also found that miR-124 was upregulated in M2 macrophages but was not induced in M1→M2 cells (**Fig. 3C, ***M* *1/M2*). Thus treatment of M1 macrophages with IL-4 did not result in either upregulation of miR-124 or conversion of M1 cells into M2 as determined by expression of phenotypical markers.

### miR-124 Contributes to the Upregulation of CD206 and Ym-1 and Downregulation of CD86, iNOS, and TNF in M2 Polarized Macrophages

We further investigated whether miR-124 expression contributed to the expression of the M2 markers in the polarized macrophages. As we mentioned earlier, overexpression of miR-124 resulted in downregulation of the M1 markers (MHC class II and CD86) and up-regulation of the M2 markers (TGF-β1, Fizz1 and Arg1) [15]. Here we investigated, whether a knockdown of miR-124 in macrophages treated with IL-4 would result in a change of their M2-indiced phenotype. As it was established earlier by other investigators and confirmed by our previous studies, the treatment of macrophages with IL-4 upregulated M2 markers including TGF-β1, Fizz1, Ym1, Arg1, CD206 and downregulated several M1 markers such as iNOS and TNF [8]. In addition to that, we found that the treatment with IL-4 upregulated miR-124, while treatment with IFN-γ/LPS upregulated miR-155 (**Fig. 3B,C**).

**Figure 4:**
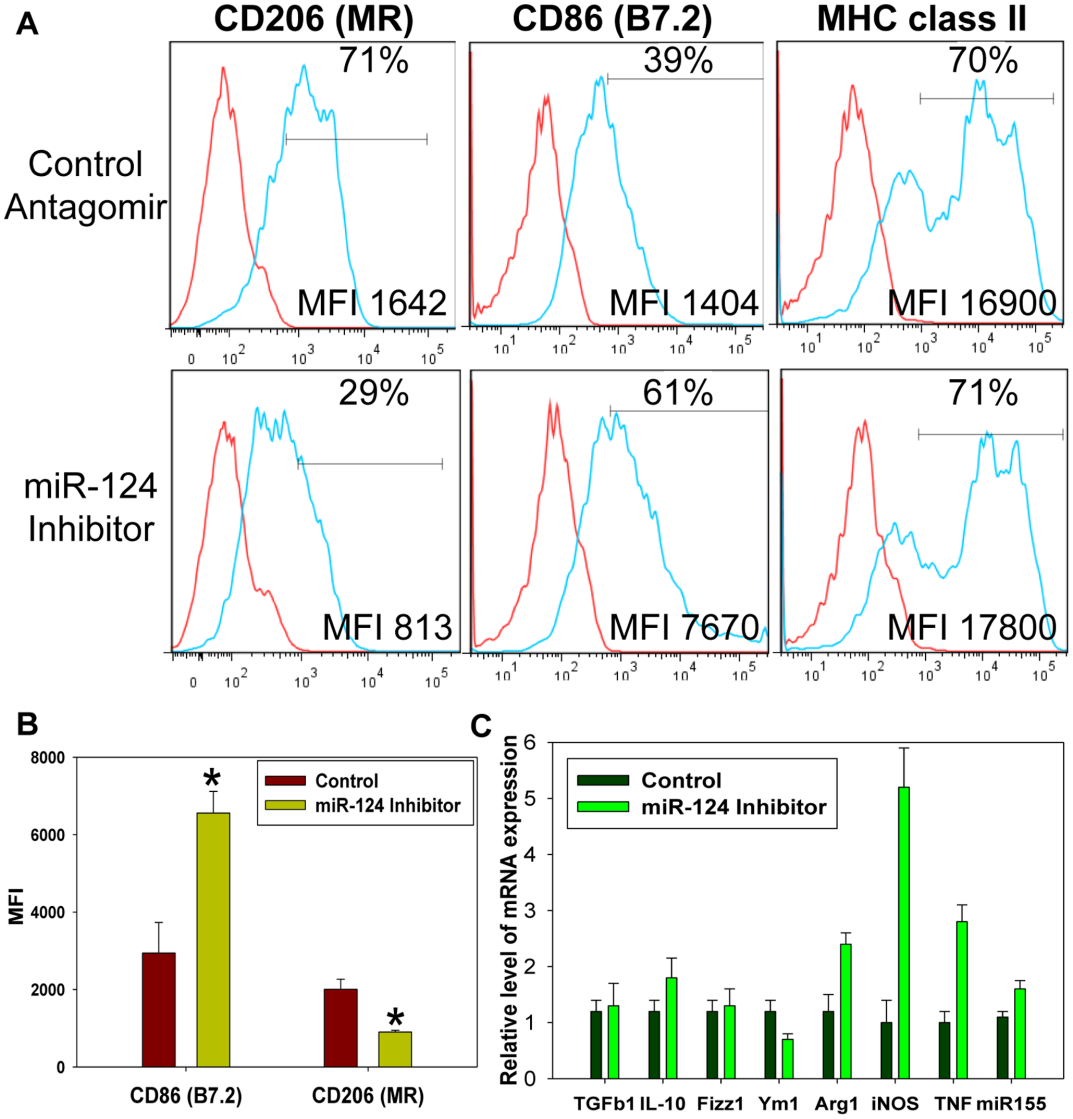
miR-124 inhibitor decreased expression of M2 markers CD206 and Ym-1 and increased expression of M1 markers CD86, iNOS, TNF, and miR-155 in the *in-vitro* polarized M2 macrophages. Peritoneal CD11b^+^F4/80^+^ macrophages were isolated and treated with IL-4 in the presence of liposomes with miR-124 inhibitor or control antagomir for 24 hours as described in *Materials and Methods*. After which, viable cells were separated using Ficoll gradient and either stained for CD206, CD86 and MHC class II and analyzed by FACS (**A, B**), or used for mRNA isolation and analysis of expression of mRNA transcripts for *TGF-β1*, *IL-10*, *Fizz1*, *Ym1*, *Arg1*, *iNOS*, *TNF*, and *miR-155* (**C**). In (**A**), the red line represents staining for isotype-matched control and the blue line - staining for surface markers. Each histogram shows overlay images. The percentage of positive cells is shown on each histogram in the upper right corner and the mean fluorescence intensity (MFI) - in the bottom right corner. The mean MFI ± S.E. of three separate experiments is shown in (**B**) (*, p<0.05) and mean ± S.E. of a triplicate is shown in (**C**).

To test the role of miR-124 in induction and maintenance of M2 phenotype, we performed the M2 polarization of the peritoneal macrophages by IL-4 in the presence of a miR-124 inhibitor (anatagomir) or control scrambled antagomir. We found that in IL-4 treated macrophages miR-124 inhibitor decreased expression of M2 markers CD206 and Ym-1 and increased expression of M1 markers CD86, iNOS, TNF and miR-155 when compared to control (**Fig. 4A–C**). The miR-124 inhibitor had a little or no effect on the expression of MHC class II (**Fig. 4A**), TGF-β1 and Fizz1 (**Fig. 4C**) in the IL-4 treated macrophages. Surprisingly, the expressions of M2 markers Arg1 and IL-10 were even enhanced in the presence of the miR-124 inhibitor (**Fig. 4C**), probably due to the compensatory redundant mechanisms of regulation of Arg1 and IL-10 expressions by IL-4. Thus miR-124 contributes to the M2 phenotype acquisition by affecting the expression of multiple markers such as CD206, Ym-1, iNOS, TNF and CD86.

### Human Intermediate CD14^+^CD16^+^ Monocytes Express High Levels of miR-124 and Exhibit Properties of M2-like Cells

**Figure 5:**
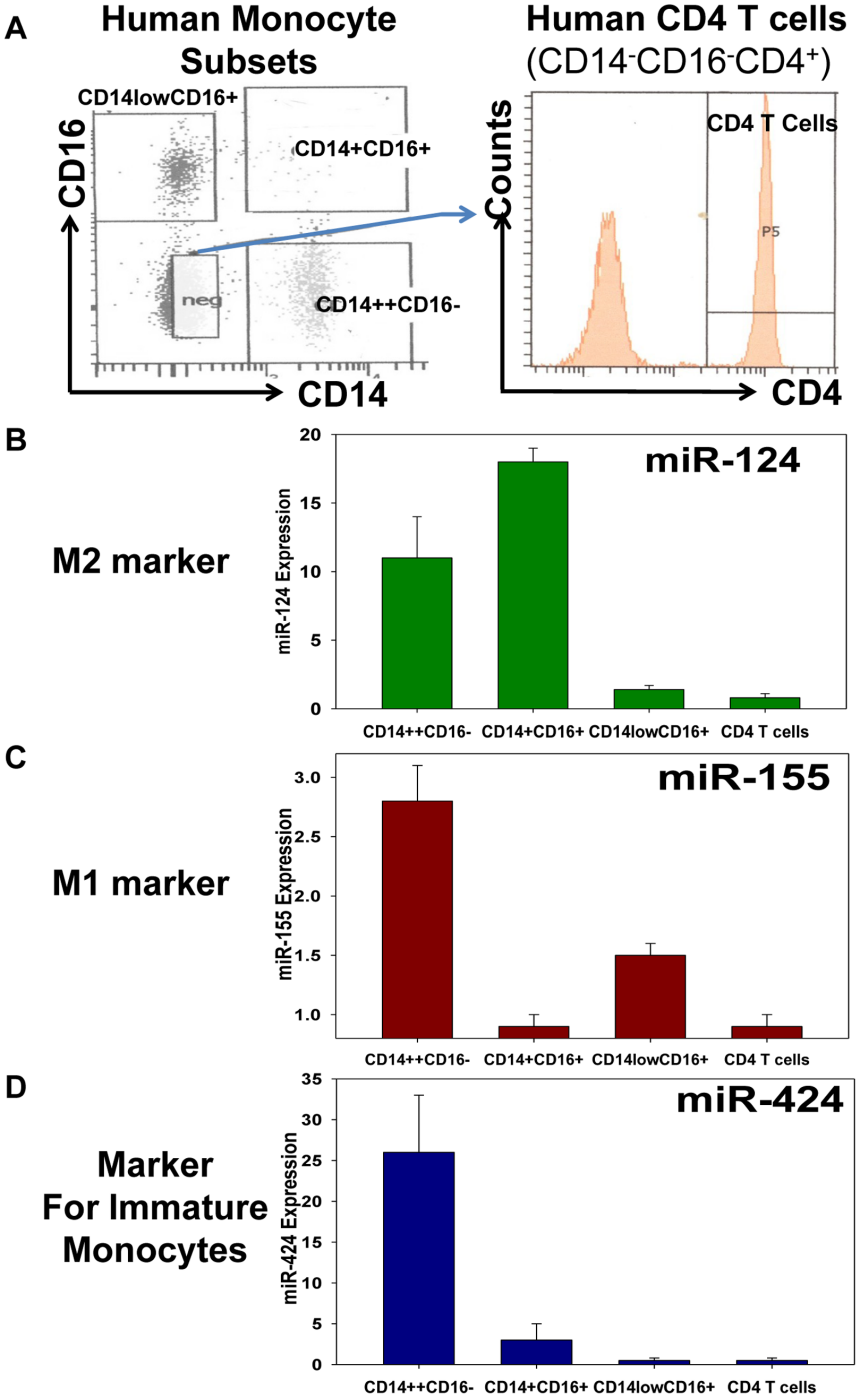
Analysis of expression of miR-124 (the M2 marker), miR-155 (the M1 marker) and miR-424 (the marker for immature monocytes) in the populations of classical (CD14^++^CD16 ^−^ **), intermediate (CD14^+^CD16^+^) and non-classical (CD14^low^CD16^+^) human monocytes.** (**A**) Mononuclear cells were isolated from the peripheral blood of healthy individuals and stained for CD14, CD16 and CD4. The populations of CD14^++^CD16^−^, CD14^+^CD16^+^, and CD14^low^CD16^+^ monocytes and CD14-CD16-CD4+ CD4 subsets were sorted by FACS and the expression of miR-124, miR-155, and miR-424 was analyzed as described in *Materials and Methods*. The mean MFI ± S.E. of three separate experiments with three healthy individuals is shown. Expression of miR-124 (**B**), miR-155 (**C**) and miR-424 (**D**) was analyzed in three sorted monocyte populations and compared to CD4 T cells. The level of expression of indicated miRNAs in CD4 T cells was used as a reference value.

It was reported that human monocytic cells are altered in the peripheral blood of patients with allergies and severe asthma leading to a selective expansion of intermediate CD14^+^CD16^+^ subset [16], [17]. We further investigated the expression of miR-124 in human peripheral blood monocytes and found that in contrast to their mouse counterparts that expressed rather low amounts of miR-124 [15], human circulating monocytes exhibited high levels of miR-124 (not shown). We then asked which subset of human monocytes expressed miR-124 and found that classical CD14^++^CD16^−^ monocytes had intermediate levels of miR-124, CD14^+^CD16^+^ monocytes contained the highest levels of miR-124, and non-classical CD14^low^CD16^+^ were virtually miR-124-negative compared to that of CD4 T cells used as a negative control (**Fig. 5A**). Since it was reported that the expression of miR-155 contributed to the phenotype of M1 cells [13] **(** **Fig. 3B** **)**, we investigated the expression of this microRNA in the aforementioned three subsets of human monocytes. The classical CD14^++^CD16^−^ monocytes expressed the highest levels of miR-155. On the other hand, CD14^+^CD16^+^ intermediate monocytes expressed miR-155 only at a background level found in CD4 T cells. Finally, non-classical CD14^low^CD16^+^ monocytes also expressed miR-155 (**Fig. 5B**). Thus, classical monocytes demonstrated the properties of dually activated M1/M2-like cells, intermediate CD14^+^CD16^+^ monocytes exhibited the properties of M2-like cells, and non-classical CD14^low^CD16^+^ monocytes those of the M1-like cells. We also used the expression of miR-424, that determines early stages of differentiation of (immature) monocytic cells [22], as a an additional control microRNA, and found that in contrast to two other subsets of CD16^+^ monocytes, classical CD14^++^CD16^−^ monocytes had high levels of the miR-424 expression. Intermediate CD14^+^CD16^+^ monocytes showed low levels of miR-424 expression and non-classical macrophages were miR-424-negative (**Fig. 5C**). Our data is in good agreement with previous studies by others indicating that classical monocytes represent a less mature population and classical and intermediate subsets are related to each other closer than to non-classical monocytes [23]. We now show that classical and intermediate subsets expressed miR-124 and miR-424 while non-classical monocytes did not, suggesting that intermediate CD14^+^CD16^+^ monocytes exhibited a more mature phenotype and properties of the M2-like cells.

## Discussion

We have shown previously that miR-124 determines the phenotype of the resident macrophages (microglia) in the CNS that exhibited properties of the M2 macrophages [8], [15]. In this study we demonstrate that miR-124 contributes to the M2 phenotype of monocytic cells of various origins from several anatomic locations. We found that miR-124 is upregulated in macrophages either through an IL-4/IL-13-dependent pathway during an allergic inflammation in the lung or independent of the IL-4/IL-13 pathway in case of the microglia.

Recent findings provided evidence that in addition to the microglia, resident macrophages in many tissues exhibit properties of the M2 cells in their default state (reviewed by Mills [6]). Our previous study established that miR-124 directly targeted CEBPα, a master transcription factor of differentiation of monocytic cells [13], [15]. We showed that miR-124 dowregulated CEBPα in microglia and macrophages resulting in the acquisition of the M2-like phenotype [15]. It was recently shown that Trib1, a molecule involved in lipid metabolism, was also required to support the M2-like phenotype (the expression of CD206, Arg1 and Fizz1) of resident macrophages in bone marrow, spleen, lungs and adipose tissue [9]. Trib1 is involved in the regulation of protein degradation by interacting with the E3 ubiquitin ligase. Interestingly, the level of CEBPα expression in Trib1 deficient mice was elevated due to the impaired degradation of this transcription factor resulting in the loss of the M2 phenotype of resident macrophages [9]. In line with our previous findings [15], the authors demonstrated that increased expression of CEBPα correlated with the loss of the M2 phenotype [9]. Thus we believe that at least in the case of resident macrophages, the downregulation of CEBPα by miR-124 or by other means (e.g. CEBPα degradation regulated by Trib1) results in skewing towards the M2-like phenotype. Metabolic changes in the adipose tissue [9], [24] or autoimmune inflammation in the CNS both resulted in a shift of balance from the M2 phenotype towards the M1 phenotype in resident macrophages [8], [15]. In the current study we show that during the allergic inflammation in the lung the balance is shifted towards the M2 phenotype, resulting in the upregulation of miR-124.

It was demonstrated that IL-4 is important for the expansion of resident macrophages and the maintenance of their M2 phenotype *in vivo* [3]. We found that miR-124 contributed to the development of this phenotype resulting in the upregulation of CD206 and downregulation of CD86. As demonstrated in the studies utilizing the Trib1-deficient mice, increased expression of CEBPα resulted in the dowregulation of CD206 [9], while we previously showed that a decrease in CEBPα expression resulted in the downregulation of CD86 [15]. Since we postulated that miR-124 targeted CEBPα [15], we hypothesized that miR-124 contributed to the upregulation of CD206 and downregulation of CD86 in IL-4 treated macrophages by targeting CEBPα. We were able to link the CEBPα-PU.1 pathway was to the regulation of the CD86 expression [15]. However both from our study and the study by Satoh et al. [9] it is not yet clear, how the downregulation of CEBPα leads to the upregulation of CD206. Future experiments will determine the particular mechanisms of regulation of CD206 expression by miR-124 and CEBPα. One of the possible alternative mechanisms of skewing macrophages towards the M2 phenotype is by the induction of the miR-124 expression utilizing the ability of miR-124 to target IL-6R and inhibit the IL-6R-STAT3 pathway, which is associated with the development of the M1 phenotype [25]. As for miR-124 it was also shown that another M2-associated microRNA, miR-223, was capable of inhibiting the STAT3 pathway [26]. Interestingly, in contrast to our previous studies when overexpression of miR-124 led to the upregulation of mRNA for TGF-β1, Fizz1 and Arg1 [15], the inhibition of miR-124 in IL-4-treated macrophages of the current study did not result in the dowregulation of expression of transcripts for TGF-β1, IL-10, Fizz1 and Arg1. We envision two possible explanations for this phenomenon. First, the miRNA inhibitors may not work efficiently enough to completely counteract the function of miR-124 in the cells. Second, there exist other redundant molecular mechanisms involved in the regulation of these markers. We favor the second possibility, since we found that the expression of Arg1 was elevated even further in the presence of the miR-124 inhibitor. One of the possible candidates that may cause the upregulation of Arg1 is miR-223, which is expressed in macrophages in the periphery but not in the CNS [15]. It was recently demonstrated that miR-223 is up-regulated by IL-4 resulting in the upregulation of Arg1 [14]. Given the fact that miR-124 negatively regulates the expression of miR-223 as shown by us previously [15], there is a possibility that inhibition of miR-124 in the IL-4 treated macrophages results in a further upregulation of miR-223 and further increase in the Arg1 expression. Thus there is a possibility that miR-223 can substitute for miR-124 in macrophages treated with miR-124 inhibitor. Finally, besides miR-124 and miR-223, other microRNAs may also contribute to M2 phenotype of macrophages further suggesting redundant mechanisms in the regulation of M2 polarization by several microRNAs [27].

Thus overexpression of miR-124 resulted in downregulation of M1 genes (CD86, TNF, iNOS) and upregulation of M2 genes (Fizz1, TGF-b1, Arg1) as we previously reported [15]. Therefore miR-124 overexpression could replace IL-4 in order to induce expression of M2 related genes and downregulate expression M1 related genes. In this study, we demonstrated that miR-124 is induced by IL-4 and inhibition of miR-124 in IL-4 treated macrophages resulted in downregulation of several M2 genes (CD206, Ym-1) and upregulation of M1 genes (CD86, iNOS, TNF, miR-155). However expression of Fizz1 and TGF-β1 were not changed and expressions of Arg1 and IL-10 were even enhanced by miR-124 inhibitor, suggesting that there are other mediators for M2 activation besides miR-124. Thus miR-124 could only partially substitute for IL-4 in knockdown experiments.

Also, *in vitro* macrophages often exhibit the properties of M1 or M2-like cells depending on the cell culture conditions. Many factors were reported to contribute to the M2-like phenotype including fetal bovine serum (FBS), M-CSF, apoptotic bodies, etc. On the other hand, GM-CSF, cell debris or serum-free media often induce the M1-like phenotype [6]. We found that BM-derived macrophages from B6 mice even in the media with FBS often exhibited the M1-like properties as determined by the MHC class II, CD86, and TNF expression [15]. Therefore, *in-vitro* experiments aiming at inducing the clearly defined M1 or M2 phenotype could be rather difficult to perform due to a high variability. We also found that induction of miR-124 in cultured bone-marrow derived macrophages depended highly on their initial state of activation and the baseline levels of expression of TNF. On the other hand, *ex-vivo* isolated peritoneal macrophages were already of the M2-like phenotype, given they were producing IL-4 (**Fig. S1A**) and were able to up-regulate miR-124 in response to the exogenous IL-4 up to 2–8 fold (**Fig. 1**). However, our *in vivo* results demonstrate the very dramatic (11-fold) upregulation of miR-124 during an allergic inflammation, indicating a role for miR-124 in alternative macrophage activation *in vivo* (**Fig. 2**).

Allergy is a rather common disease of the modern world, which is likely to be connected to the shift of the M1/M2 polarization balance in the lung macrophages (reviewed by Mills [6] ). It was demonstrated that allergic inflammation is associated with the M2 activation of macrophages *in vivo* [28], [29]. The importance for IL-4 and IL-13 for the development of allergic inflammation was clearly demonstrated in mouse models using IL-4 and IL-4/13R deficient mice [21], [30]. Moreover, IL-4/13R-STAT6 signaling pathway was essential for both the induction of M2 phenotype in the lung macrophages and the development of an allergic lung inflammation [21]. Several microRNAs were recently shown to become upregulated during an allergic lung inflammation. miR-146 and miR-21, which are involved in the macrophage activation and the resolution of inflammation, respectively [13], were shown to be associated with allergic responses [31], [32], so as was miR-223 which was recently shown to contribute to the M2 phenotype acquisition [14]. However, miR-223 is prominently expressed in granulocytes and is not fully M2-specific [33]. Our study clearly demonstrates that miR-124 may be used as an macrophage-specific M2 marker, which is significantly upregulated in macrophages during an allergic inflammation in mouse model.

In addition to the expression of miR-124 in lung macrophages during an allergic lung inflammation, miR-124 could potentially serve as a marker for changes of M1/M2 balance in the periphery. We found that human monocytes from the peripheral blood of healthy individuals expressed miR-124 with the highest level of expression in the subset of the intermediate CD14^+^CD16^+^ monocytes. It was reported that this intermediate CD14^+^CD16^+^ subset (same subset is also referred to as CD14^++^CD16^+^ or CD14^high^CD16^+^) was expanded in patients with allergies [16] and asthma [17] and expressed a number of M2 surface markers such as CD163, CX3CR1, IL-4R, TGF-β1 and IL-10 [17], [34]–[36]. The classical CD14^+^CD16^−^ monocytes (or same subset of CD14^++^CD16^−^ or CD14^high^CD16^−^ cells as described in other publications) expressed both miR-124 and miR-155 (miR-155 is associated with the M1 phenotype of the pro-inflammatory macrophages in pathology [13]) and exhibited the properties of dually activated M1/M2-lke cells (**Fig. 5**) [23], [37] similarly to the macrophages that migrated into the CNS from the periphery in mice with neuroinflammation [8]. The non-classical CD14^low^CD16^+^ subset (also referred to as CD14^+^CD16^++^ or CD14^dim^CD16^+^ in other publications) exhibited the properties of the M1-like cells by expressing miR-155, TNF, IL-6, ROS, TLR4 and high levels of CCR2 [23], [35], [38], [39]. The expression profile of the M1 and M2-specific markers as well as the miRNA expression patterns in these three subsets of human monocytes are summarized in **Table 2**.

**Table 2:** Overview of the M1 and M2 phenotypic markers and microRNA expression in the populations of classical (CD14^++^CD16^−^), intermediate (CD14^+^CD16^+^) and non-classical (CD14^low^CD16^+^) monocytes and their involvement in various pathological conditions^1^.

Monocyte Subset (Alternative Identification)	Classical CD14^++^CD16^−^ (CD14^+^CD16^−^) (CD14^high^CD16^−^)	Intermediate CD14^+^CD16^+^ (CD14^++^CD16^+^) (CD14^high^CD16^+^)	Non-classical CD14^low^CD16^+^ (CD14^+^CD16^+^) (CD14^dim^CD16^+^)
**Proposed type of activation/polarization**	**M1/M2-like**	**M2-like**	**M1-like**
**Maturation**	Immature	Mature	Very mature
**MicroRNA expression**	**miR-155; miR-124 (intermediate); miR-424 (high)**	**miR-124 (high); miR-424 (low)**	**miR-155**
**M1 Markers**	miR-155; MHC class II (low); CD86(low); CCR2 (intermediate)	MHC class II; CD86 (intermediate); CCR2 (low)	miR-155; MHC class II; CD86 (high); CCR2 (high); TLR4; TNF; ROS; Stimulation of T cells
**M2 Markers**	miR-124 (intermediate); CD163 (intermediate); IL-10	miR-124 (high); CD163 (high);IL-10; CD206; IL-4R; CX3CR1	−
**Conditions for expansion** **(increase in percentage)**	GM-CSF administration	Allergy, Asthma	Inflammation, Infection, Autoimmunity: TB^2^; HIV; SIV; Sepsis; Atherosclerosis; RA; T1D; IBD; MS(?)

^1^ See explanations and references in the text.

^2^ Abbrevations: TB, tuberculosis; HIV, human immunodeficiency virus; MS, multiple sclerosis SIV, simian immunodeficiency virus; RA, rheumatoid arthritis; T1D, type 1 diabetis; IBD, inflammatory bowel disease.

At the baseline, intermediate CD14^+^CD16^+^ (also referred to as CD14^++^CD16^+^) subset expressed the highest level of transcripts for TGF-β1 when compared to other two monocyte subsets [36]. This confirms our observation that intermediate CD14^+^CD16^+^ cells have M2-like phenotype. On the other hand, unstimulated non-classical CD14^low^CD16^+^ cells were potent stimulators of T cells when compared to other two subsets of intermediate and classical monocytes. This suggests that non-classical monocytes exhibit M1-like phenotype [35], which confirm our observation in **Fig. 5** **.** Upon LPS stimulation, intermediate CD14^+^CD16^+^ cells were the only population that produce IL-10, whereas non-classical CD14^low^CD16^+^ were the main producers of TNF [35]. These data also confirmed our observation shown in **Fig. 5** that CD14^+^CD16^+^ cells have M2-like phenotype, whereas CD14^low^CD16^+^ cells have properties of M1 cells. Recently it was reported that in patients with multiple sclerosis (MS), an autoimmune disease associated with Th1 autoimmune cells and shifting balance towards M1, the expression of miR-124 was downregulated and expression of miR-155 was upregulated in whole monocyte population [40]. Thus upregulation of miR-155 and downregulation of miR-124 could represent expansion of non-classical monocytes during autoimmune diseases such as MS (**Table 2**).

The miRNA expression data we obtained, combined with the phenotypical markers gives us a rather interesting perspective since CD14^low^CD16^+^ non-classical monocytes are expanding during inflammation, infection and autoimmunity, whereas the functions of the intermediate CD14^+^CD16^+^ monocytes are not well known and these cells could hypothetically contribute to allergy [38], [41]. The classical monocytes were reported to be less mature [36], but our findings demonstrate that they also exhibit a dually activated M1/M2 phenotype (**Table 2**). Thus miR-124 in monocytic cells appears to be a useful marker for the M2-like macrophages and the overexpression or inhibition of miR-124 in macrophages may be beneficial for future therapeutic approaches to alter the balance towards M2 or M1.

## Materials and Methods

### Mice

B6 (C57BL/6), BALB/c, IL-4 (B6-Il4^tm1Nnt^/J), IL-4/13R (BALB/c-Il4ra^tm1Sz^/J) and STAT6 (B6-*Stat6^tm1Gru^*/J) deficient mice were purchased from Jackson Laboratories (Bar Harbor, ME). Animals were housed in the Harvard Institutes of Medicine specific pathogen free animal facility. All animal protocols were approved by the Harvard Medical School Institutional Animal Care and Use Committee and Chinese University of Hong Kong Animal Ethics Committee. For experiments that involved pain and distress the appropriate anesthetic and analgesic drugs were used. Euthanasia was performed by using carbon dioxide.

### Antibodies and Reagents

The fluorochrome-conjugated or biotinylated antibodies for mouse CD11b, F4/80, MHC class II, CD86, and CD206 were purchased from BD Biosciences, eBioscience, and Biolegend (all form San Diego, CA). Anti-IL-4 antibodies and cytokines IFN-γ, IL-4, IL-13, TGF-β1 and IL-10 were purchased from R&D Systems (Minneapolis, MN). The fluorochrome-conjugated antibodies for human CD4, CD14 and CD16 were purchased from BD Biosciences (San Diego, CA). miR-124 antagomir inhibitor or control scrambled antagomir were purchased from Exiqon Inc (Woburn, MA). LPS, chicken ovalbumin (OVA), and alum adjuvant were purchased from Sigma (St. Louis, MO). For all experiments, OVA protein was depleted of endotoxin (LPS) using Affinity-Pak™ Detoxi-Gel™ columns (Pierce Biotechnology, Rockford, IL) according to the manufacturer’s instructions. IL-4 complexes (IL-4c) were prepared by mixing IL-4 and anti-IL-4 antibodies and injected i.p. daily for 3 days as described previously [3].

### OVA-induced Allergic Inflammation

BALB/c and BALB/c-IL4/13R KO, or B6 and B6-IL4 KO mice were injected i.p. three times weekly with OVA combined with the adjuvant for the sensitization and then challenged intranasally with the OVA solution similarly as described [29]. For the sensitization, 20 µg of OVA were adsorbed onto 2.25 mg of alum adjuvant (Pierce Biotech, Rockford, IL) by mixing for one hour at room temperature in a total volume of 200 µl endotoxin-free PBS (Sigma). One week after the last sensitization, OVA challenge was initiated by instilling 25 µg OVA in 25 µl endotoxin-free PBS into each nostril daily for five consecutive days. During all experimental procedures, mice were anesthetized by 2.5% isoflurane (Baxter Healthcare, Deerfield, IL) in a closed chamber using a VetEquip Inhalation Anesthesia System (Pleasanton, CA). For experiments, mice were sacrificed 24 hours after the last challenge.

### Cell Lines

RAW 264.7 cell line was purchased from ATCC (Manassas, VA) and maintained in DMEM media supplemented with 5% FBS. RAW264.7 cells were incubated with IL-4 at a range of concentrations (10–200 ng/ml) for 0.5, 4 and 24 hours.

### Bronchoalveolar Lavage (BAL)

The lungs of sacrificed animals were lavaged by cannulating the trachea and gently injecting/recovering (5X) of 0.8 ml of a pre-warmed (37°C) lavage fluid (PBS w/o Mg^+2^ and Ca^+2^). The BAL fluid was combined in an ice-cold 15 ml Falcon tube containing 100 µl of FBS (Invitrogen, Carlsbad, CA), and then passed through a 70 µm nylon cell strainer filter (BD Biosciences) to remove cell debris. Filtered BAL fluid was centrifuged at 300 g, 4°C for 10 minutes and the resulting cell pellet was washed twice using 0.5 ml of PBS and then re-suspended in 0.5 ml of FACS buffer (HBSS with 10 mM HEPES, pH 7.4, 2% FBS).

### Cell Isolation and Culture

For bone marrow (BM)-derived macrophages, BM was isolated from the 4–6 weeks old B6 mice; after erythrocyte lysis, mononuclear cells were incubated with M-CSF (10 ng/ml) in DMEM media (ATCC) supplemented with 10% FBS for 5 days as described [15]. The medium was changed every 3 days. Peritoneal macrophages were isolated from unmanipulated B6 mice by means of a peritoneal lavage with PBS as described earlier [15], [20] and magnetic beads-sorted population of F4/80^+^CD11b^+^ cells was used for polarization towards the M1 or M2 phenotype. For the M1 or M2 polarization, *ex-vivo* isolated and pre-sorted peritoneal or cultured BM-derived macrophages were incubated with IFN-γ (200 ng/ml) and LPS (200 ng/ml); or IL-4 (50–100 ng/ml), or IL-13 (50–100 ng/ml), or TGF-β1 (100–200 ng/ml), or IL-10 (100–200 ng/ml) for 24 hours accordingly, as described earlier [8], [20].

For the isolation of microglia from the normal CNS, mice were perfused intracardially with PBS prior to the dissection of the brain and spinal cord, which were then homogenized; mononuclear cells were isolated from the homogenates using 40%/70% Percoll gradients. FACS analysis demonstrated a 94–96% purity of the CD11b^+^CD45^low^ microglial cells in the preparation of the CNS mononuclear cells as was described previously in our studies [8], [42], [43].

For the isolation of alveolar macrophages, BAL cells from each mouse were sorted by FACS using FSC/SSC/FL-1 gating strategy, which is based on the selection of cells with an increased size, medium granularity and high autofluorescence that correspond to the population of alveolar macrophages [29].

For the isolation of lung resident (interstitial) macrophages, lungs after BAL were digested for one hour at 37°C with a Collagenase and DNase mix solution (Sigma). The digested material was than filtered through a 70 µm nylon mesh cell strainer (BD Falcon) to prepare cell suspensions and lung macrophages were sorted by using anti-mouse CD11b magnetic beads (Miltenyi Biotech Inc.) according to instructions provided by the kit.

In all our conditions for various mouse strains, we isolated sufficient amount of macrophages from lungs: 0.5–0.9×10^6^/mouse for alveolar macrophages and 1–3×10^6^/mouse for interstitial macrophages to perform analysis of miR-124 expression by real-time RT-PCR.

Human mononuclear cells from peripheral blood of healthy individuals were isolated by Ficoll*-*Hypaque Density Media (Sigma) and the cells were stained for CD4, CD14 and CD16 in FACS buffer after blocking with FcR blocking antibodies (BD Biosciences) and the population of mononuclear cells was determined using the FSC/SSC mononuclear gate for the analysis of the CD14, CD16 and CD4 surface markers expression. Use of normal human peripheral blood mononuclear cells from anonymous healthy individuals for *in vitro* experiments was approved by the institutional review boards of the Brigham and Women’s Hospital and all healthy subjects provided written informed consent prior to enrollment into study. The data from human blood samples was obtained and analyzed without records of any personal information to ensure complete anonymity.

In all experiments, isolated and sorted cells were immediately centrifuged at 300 g for 10 minutes at 4°C, the supernatant was carefully decanted, the cell pellet was snap frozen on dry ice and stored at −80°C for the subsequent RNA isolation and miRNA expression analysis [44].

### Transfection of Macrophages with miR-124 Inhibitor

The miR-124 inhibitor (LNA-containing antisense oligonucleotide) or control scrambled antagomir (both from Exiqon) were used for *in vitro* transfections of the peritoneal macrophages cultured in the presence of IL-4 for 24 h. For transfection, miR-124 inhibitor was complexed with Lipofectamin 2000™ (Invitrogen) as described previously [15], [45].

### Flow Cytometry and Cell Sorting

The 1–3–color flow cytometry analysis was conducted in the Flow Cytometry sorting core facility following standard procedures. Staining of the cells for their surface markers was performed after the FcR blocking (BD Biosciences) in FACS buffer on ice for 20 minutes as described earlier [44]. The Flow cytometry analysis was conducted on the LSR II and LSR-Fortessa Cytometers, and the cell sorting was performed on the FACSAria Cytometer (all from BD Biosciences). Sorting purities were 98–100% as determined on the FACSAria sorter by reanalyzing the sorted populations [44].

### Analysis of miRNA and pri-miRNA Expression

For the analysis of miR-124, miR-155, and miR-424 expression in the populations of monocytic cells, total RNA was isolated using miRNeasy Mini Kit (Qiagen), and real-time RT-PCR analyses were carried out using the TaqMan miRNA assays and the proper mouse and human primer and probe sets (Applied Biosystems). The relative expression was calculated by using the ΔC_T_ method and normalized to the uniformly expressed snoRNA55 (Applied Biosystems) as described earlier [44]. For analysis of miRNA expression, we first normalized our data to housekeeping short non-coding RNA snoRNA55 and then normalized to reference sample (defined as 1.0). In all our experiments snoR55 was detectable after 20–25 cycles of amplification. Pri-miR124.1, pri-miR-124.2 and pri-miR-124.3 were analyzed using the mm03307218 (pri-mmu-124-1), mm03307226 (pri-mmu-124-2), and mm03306225 (pri-mmu-124-3) primers from Life Technologies. All qRT-PCRs were performed in triplicates, and the data are presented as mean ± standard deviations (S.D.) or standard errors (S.E.).

### Analysis of mRNA Expression

For the analysis of the mRNA expression for TGF-β1 (forward primer, 5′-CAGAGCTGCGCTTGCAGAG; reverse primer, 5′-GTCAGCAGCCGGTTACCAAG); Arg1 (forward primer, 5′-CTTGGCTTGCTTCGGAACTC; reverse primer, 5′- GGAGAAGGCGTTTGCTTAGTTC); Fizz1 (forward primer, 5′-GCCAGGTCCTGGAACCTTTC; reverse primer, 5′-GGAGCAGGGAGATGCAGATGAG); IL-4 (forward primer, 5′-GTCTGCATCAAGACGCCATG; reverse primer, 5′-CGTTGCTGTGAGGACGTTTG); **iNOS** (forward primer, 5′-ACCCACATCTGGCAGAATGAG; reverse primer 5′-AGCCATGACCTTTCGCATTAG); TNF (forward primer, 5′- AGCCGATGGGTTGTACCTTG; reverse primer, 5′- GTGGGTGAGGAGCACGTAGTC); Ym1/Chi3l3 (Mm00657889_mH; purchased from Life Technologies); and IL-10 (Mm00439614_m1; purchased from Life Technologies) real-time RT-PCR analyses were carried out by using TaqMan and the relative expression was calculated by using the ΔC_T_ method and normalized to the GADPH housekeeping gene (forward primer, 5′-ATGACCACAGTCCATGCCATC; reverse primer, 5′-GAGCTTCCCGTTCAGCTCTG). All qRT-PCRs were performed in triplicate, and the data are presented as mean ± standard deviations (S.D.).

### Statistical Analysis

Student’s t-test and Mann Whitney u-test were used to validate the significance of the observed differences. A p-value of less than 0.05 was considered statistically significant.

## Supporting Information

Figure S1 Comparison of IL-4 (A) and TNF (B) expression at M0 state in microglia, peritoneal macrophages and bone-marrow (BM)-derived macrophages in two separate experiments. BM-derived and peritoneal macrophages were obtained from WT B6 mice as in Fig. 1, while microglial cells were isolated from the CNS of WT B6 mice as described in *Materials and Methods*. RNA was isolated and the expression of mRNA transcripts for IL-4 and TNF was analyzed by real-time RT PCR as described in *Materials and Methods*. Results are shown in duplicates (shown as “Duplicate 1” and “Duplicate 2”) for two representative experiments indicated as ‘#1’ or ‘#2’. Abbreviations: MG, microglia; Perit.MPh, peritoneal macrophages, BM-MPh, bone marrow derived macrophages.
(TIF)

Figure S2 **Analysis of expression of miR-124 in the** ***ex-vivo*** **isolated microglia from the CNS of healthy B6-WT, B6-IL-4 (B6-IL-4 KO) and B6-STAT6 (B6-STAT6 KO) deficient (knock out) mice, or BALB/c-WT and BALB/c-IL-4/13R (BALB/c-IL-4/13R KO) deficient mice.** Microglia were isolated as described in *Materials and Methods* and the expression of miR-124 was analyzed as in Fig. 1. The data is representative of three separate experiments with Mean ± S.E. of triplicate shown.
(TIF)
