# Supplementary figures and images for: IL-4/IL-13-Dependent and Independent Expression of miR-124 and Its Contribution to M2 Phenotype of Monocytic Cells in Normal Conditions and during Allergic Inflammation
Source: PLoS One. 2013 Dec 16;8(12):e81774. doi: 10.1371/journal.pone.0081774 (PMC3864800; doi:10.1371/journal.pone.0081774)

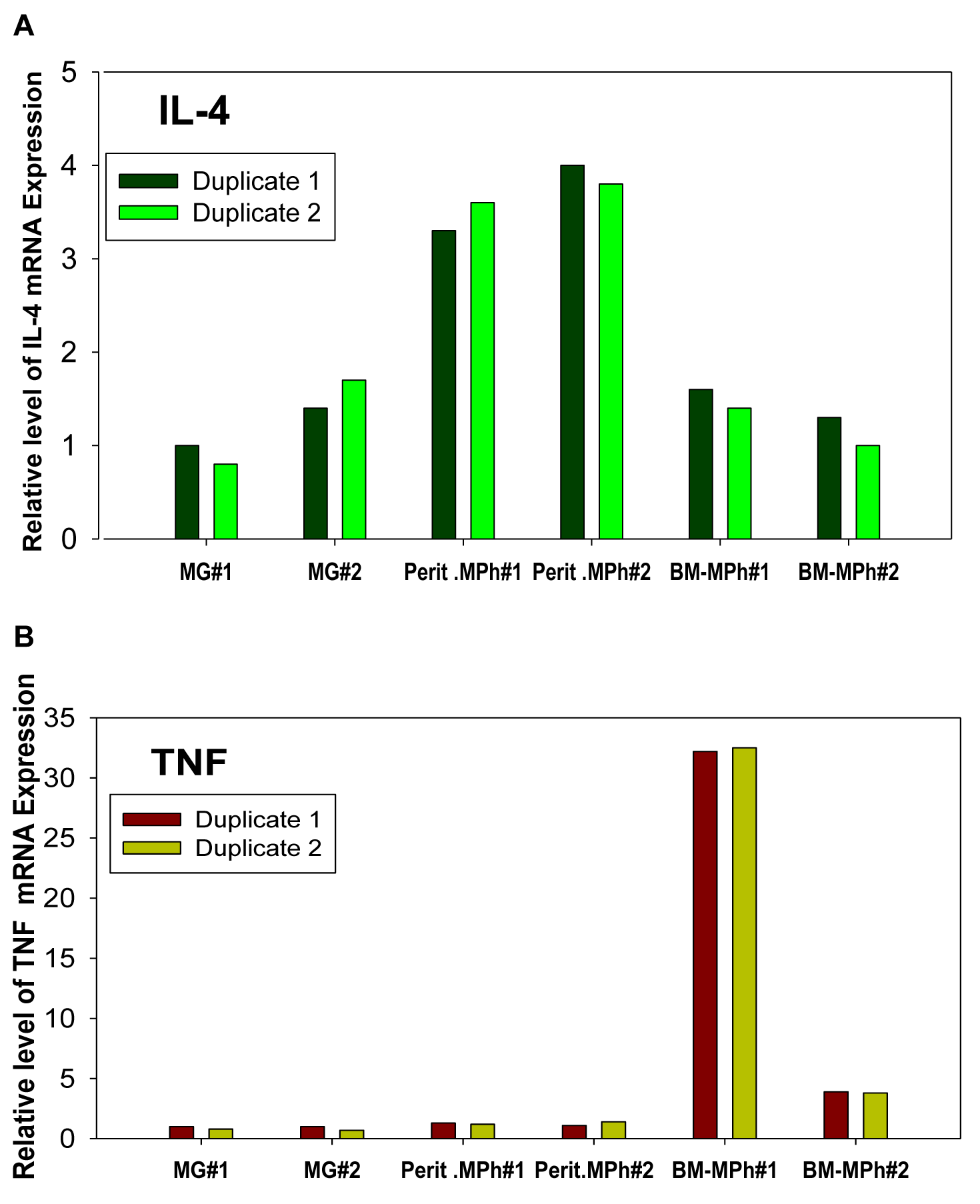

Supplement: Figure S1 — Comparison of IL-4 (A) and TNF (B) expression at M0 state in microglia, peritoneal macrophages and bone-marrow (BM)-derived macrophages in two separate experiments. BM-derived and peritoneal macrophages were obtained from WT B6 mice as in Fig. 1, while microglial cells were isolated from the CNS of WT B6 mice as described in Materials and Methods. RNA was isolated and the expression of mRNA transcripts for IL-4 and TNF was analyzed by real-time RT PCR as described in Materials and Methods. Results are shown in duplicates (shown as “Duplicate 1” and “Duplicate 2”) for two representative experiments indicated as ‘#1’ or ‘#2’. Abbreviations: MG, microglia; Perit.MPh, peritoneal macrophages, BM-MPh, bone marrow derived macrophages. (TIF) [file pone.0081774.s001.tif]

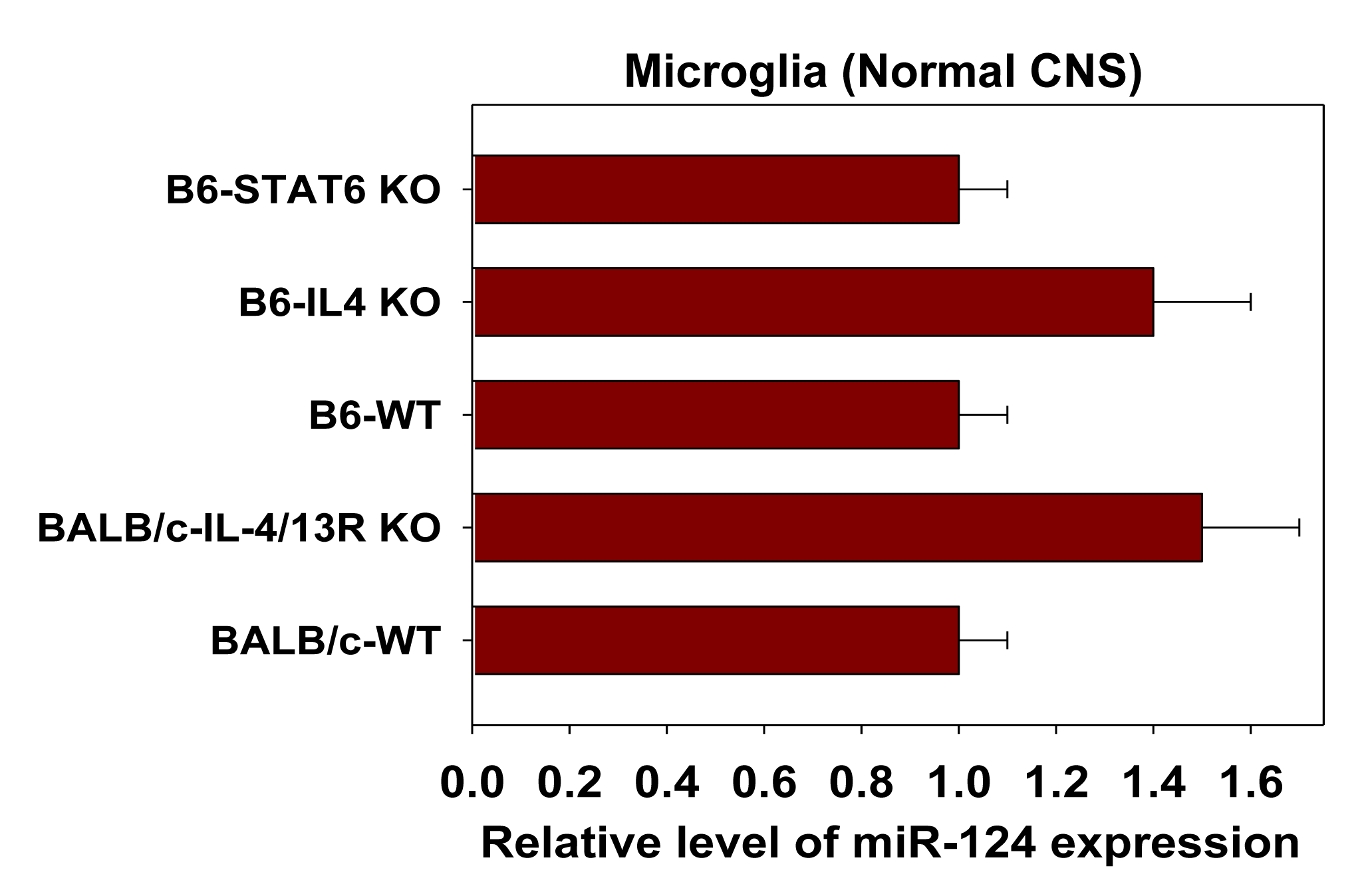

Supplement: Figure S2 — Analysis of expression of miR-124 in the ex-vivo isolated microglia from the CNS of healthy B6-WT, B6-IL-4 (B6-IL-4 KO) and B6-STAT6 (B6-STAT6 KO) deficient (knock out) mice, or BALB/c-WT and BALB/c-IL-4/13R (BALB/c-IL-4/13R KO) deficient mice. Microglia were isolated as described in Materials and Methods and the expression of miR-124 was analyzed as in Fig. 1. The data is representative of three separate experiments with Mean ± S.E. of triplicate shown. (TIF) [file pone.0081774.s002.tif]
